# Pharmacological Targeting of the RAGE-NFκB Signalling Axis Impedes Monocyte Activation under Diabetic Conditions through the Repression of SHP-2 Tyrosine Phosphatase Function

**DOI:** 10.3390/cells12030513

**Published:** 2023-02-03

**Authors:** Marc Dorenkamp, Madina Nasiry, Dilvin Semo, Sybille Koch, Ivonne Löffler, Gunter Wolf, Holger Reinecke, Rinesh Godfrey

**Affiliations:** 1 Vascular Signalling, Molecular Cardiology, Department of Cardiology I—Coronary and Peripheral Vascular Disease, Heart Failure, University Hospital Münster, 48149 Münster, Germany; 2 Department of Internal Medicine III, University Hospital Jena, 07743 Jena, Germany

**Keywords:** SHP-2, PTPN11, monocytes, NFκB, RAGE, methylglyoxal, T2DM, atherosclerosis

## Abstract

Monocytes play a vital role in the development of cardiovascular diseases. Type 2 diabetes mellitus (T2DM) is a major CVD risk factor, and T2DM-induced aberrant activation and enhanced migration of monocytes is a vital pathomechanism that leads to atherogenesis. We recently reported the upregulation of SHP-2 phosphatase expression in mediating the VEGF resistance of T2DM patient-derived monocytes or methylglyoxal- (MG, a glucose metabolite and advanced glycation end product (AGE) precursor) treated monocytes. However, the exact mechanisms leading to SHP-2 upregulation in hyperglycemic monocytes are unknown. Since inflammation and accumulation of AGEs is a hallmark of T2DM, we hypothesise that inflammation and AGE-RAGE (Receptor-for-AGEs) signalling drive SHP-2 expression in monocytes and blockade of these pathways will repress SHP-2 function. Indeed, monocytes from T2DM patients revealed an elevated SHP-2 expression. Under normoglycemic conditions, the serum from T2DM patients strongly induced SHP-2 expression, indicating that the T2DM serum contains critical factors that directly regulate SHP-2 expression. Activation of pro-inflammatory TNFα signalling cascade drove SHP-2 expression in monocytes. In line with this, linear regression analysis revealed a significant positive correlation between TNFα expression and SHP-2 transcript levels in T2DM monocytes. Monocytes exposed to MG or AGE mimetic AGE-BSA, revealed an elevated SHP-2 expression and co-treatment with an NFκB inhibitor or genetic inhibition of p65 reversed it. The pharmacological inhibition of RAGE was sufficient to block MG- or AGE-BSA-induced SHP-2 expression and activity. Confirming the importance of RAGE-NFκB signalling in regulating SHP-2 expression, the elevated binding of NFκB to the SHP-2 promoter—induced by MG or AGE-BSA—was reversed by RAGE and NFκB inhibition. Besides, we detected elevated RAGE levels in human and murine T2DM monocytes and monocytes exposed to MG or AGE-BSA. Importantly, MG and AGE-BSA treatment of non-T2DM monocytes phenocopied the aberrant pro-migratory phenotype of T2DM monocytes, which was reversed entirely by either SHP-2- or RAGE inhibition. In conclusion, these findings suggest a new therapeutic approach to prevent accelerated atherosclerosis in T2DM patients since inhibiting the RAGE-NFκB-SHP-2 axis impeded the T2DM-driven, SHP-2-dependent monocyte activation.

## 1. Introduction

Cardiovascular disease (CVD) is a chronic inflammatory disease that is the leading cause of morbidity and mortality worldwide. Diabetes is a significant independent risk factor for CVD, with diabetic and pre-diabetic patients accounting for almost 65% of all CVD deaths [1,2]. Monocyte activation is a significant determinant in the development of both coronary (CAD) and peripheral artery disease (PAD) [3,4]. Monocytes are involved in all the stages of atherosclerosis development, potentiating inflammatory responses during early plaque development and initiating the breakdown and rupture of the fibrous cap leading to myocardial infarction [5]. During the pathogenesis of diabetes-accelerated atherosclerosis, enhanced transmigration of monocytes across the endothelium has been recognised as a critical process [6]. Monocyte activation results in accelerated random motility, termed “chemokinesis”. Induction of chemokinesis in monocytes increases the likelihood of them getting tethered and roll on endothelial cells and eventually transmigrating [7]. Hyperlipidemic [8] and diabetic [9] environments are reported to induce chemokinesis of monocytes, consistent with the accumulated monocytes/macrophages in the albumen under these conditions. 

Methylglyoxal (MG) is a reactive metabolite primarily formed as a byproduct during glycolysis [10]. MG reacts directly with lysine and arginine residues on proteins to form advanced glycation end products (AGE) that compromise their function. In vitro, ex vivo or in vivo administration of MG to mice or cultured cells mimic pathological changes typically associated with diabetes [11,12]. Recently, MG has been proposed as a root cause of T2DM as elevated MG levels alone were sufficient—independent of insulin and glucose levels—to induce T2DM initiation and progression [13]. MG induces dysfunction in vascular cells, including endothelial cells [14] and smooth muscle cells [15]. On the contrary, MG activates monocytes and macrophages [9,16]. In particular, monocyte activation and random motility-induced VEGF resistance of T2DM monocytes are MG-dependent [9].

SH2 domain-containing tyrosine phosphatase 2 (SHP-2/PTPN11) is a ubiquitously expressed cytosolic protein tyrosine phosphatase (PTP) involved in the physiological control of various cytokine and growth-factor-induced signal transduction processes [17]. However, it is also known to get activated in several pathological conditions leading to impaired signalling-associated disease initiation and progression [18]. Even though SHP-2 is mainly associated with oncological manifestations [19], several studies on the role of SHP-2 in diabetes and atherosclerosis pathology have recently been reported [9,20,21]. Therefore, therapeutic targeting of SHP-2 is beneficial in cancer and other inflammatory conditions [22]. However, given the importance of SHP-2 in regulating physiological signalling processes, a more precise strategy to inhibit SHP-2 function—devised based on the specific pathological context in question—is necessary. 

In order to identify better strategies to inhibit SHP-2 activity, the potential mechanisms through which the functional activation of SHP-2 occurs in monocytes in the diabetic environment were explored in this study. The experiments revealed that in the MG-associated diabetic environment, the receptor for advanced glycation end products (RAGE)-NFκB axis drives SHP-2 expression in monocytes. Blockade or induction of RAGE signalling using different approaches blunted or accelerated SHP-2 expression upregulation. Our findings reveal the proof of concept of the feasibility of blunting SHP-2 expression in T2DM conditions by blocking RAGE—the upstream molecule with no significant physiological function—to reverse monocyte activation.

## 2. Materials and Methods

### 2.1. Materials

#### 2.1.1. Reagents

Methgylglyoxal, Glucose, FBS, Penicillin/Streptomycin were from Sigma Aldrich (Darmstadt, Germany). NFκB activation inhibitor I, NFκB activation inhibitor II (JSH23), RAGE inhibitor FPS-ZM1, RAGE antagonist peptide (RAP) and AGE-BSA were obtained from Calbiochem (Darmstadt, Germany). SHP-2 inhibitors NSC 878777 and SHP099 were brought from Tocris Bioscience (Wiesbaden-Nordenstadt, Germany) and Cayman Chemical (Tallinn, Estonia), respectively. The protein tyrosine phosphatase activity assay kit was from Promega (Walldorf, Germany). All the primers for qPCR were customer synthesised from Sigma Aldrich (Darmstadt, Germany). Primer sequences are described in Supplementary Table S2. Antibodies used for the WB and FACS are mentioned in the methods section. 

#### 2.1.2. Clinical Cohorts and Ethical Approval

Blood samples were obtained over 15 months from 24 individuals with type 2 diabetes (*n* = 12) or non-diabetic individuals (*n* = 12) visiting the Department of Cardiology I, University Hospital Münster, Münster, Germany. Smokers and individuals with infections and with anti-inflammatory therapy were excluded. The local ethics committee approved this study at Münster University Hospital, Münster, Germany. All the T2DM patients and non-T2DM individuals provided written informed consent to participate in the study. The ethical permission number is 2011-612-f-S. The study conforms to the declaration of Helsinki.

### 2.2. Methods

#### 2.2.1. Isolation of Primary Human and Murine Monocytes

Monocytes were isolated from thrombocyte reduction filters obtained from venous blood of healthy individuals as previously described [9] using Magnet assisted cell sorting (MACS) using negative selection using Monocyte Isolation Kit II human from Miltenyi Biotec (Bergisch Gladbach, Germany). The study was approved by the scientific and ethics committee of the University of Münster and conforms to the principles of the Declaration of Helsinki. Written informed consent was obtained from all donors by the blood bank, and thrombocyte reduction filters were provided anonymously without sharing personal and detailed information. Bone marrow Ly6C^+^ monocytes cells from the tibia and fibula were extracted by flushing with PBS-BSA solution. After erythrolysis, the cells were subjected to magnet-assisted cell sorting (MACS) according to the manufacturer’s instructions using the Monocyte Isolation Kit (BM) from Miltenyi Biotec (Bergisch Gladbach, Germany). The purity of isolated cells was confirmed by FACS and they were around 98% pure. 

#### 2.2.2. Animals

The T2DM mice model db/db (B6.Cg-Dock7m Leprdb/++/J) mice and non-diabetic Wt mice (Jackson Laboratory, Bar Harbor, ME, USA) were used in this study. 8–12 weeks-old mice were used for the experiments and the monocytes were isolated from the bone marrow. The animals were housed in a temperature-controlled environment and had free access to water and a regular diet (65% carbohydrate, 11% fat, 24% protein). All animal studies were carried out under the German animal protection law and with the European Communities Council Directive 86/609/EEC and 2010/63/EU to protect animals used for experimental purposes. All experiments were approved by the Local Institutional Animal Care and Research Advisory Committee and permitted by the local authority.

#### 2.2.3. Cell Culture and Treatment

Primary human monocytes and THP-1 monocytic cells (acquired from Leibniz Institute DSMZ—German Collection of Microorganisms and Cell Cultures) were maintained in RPMI-1640 medium with +L-Glutamine, −D-Glucose (Thermo Scientific, Karlsruhe, Germany) supplemented with 5 mM Glucose, 10% fetal bovine serum (FBS) and 1% Penicillin/Streptomycin. For migration experiments and signalling studies, cells were starved for 2–4 h in FBS free medium. Monocytes were kept in an incubator at 37 °C and 5% CO_2_. 

#### 2.2.4. Monocyte Chemokinesis Analysis

Monocyte chemokinesis was studied using a transwell system with 5 µm-diameter pores (Sarstedt, Nümbrecht Germany). Monocytes were treated with 300 µM MG for 24 h with and without inhibitors. After that, monocytes were added to the wells containing the transwell inserts and incubated at 37 °C in 5% CO_2_ for 8–12 h. Cells transmigrated through the pores into the lower chamber were counted and imaged using Countess II FL Automated Cell Counter (Invitrogen, Karlsruhe, Germany). Monocyte chemokinesis was also studied with the modified 48-well Boyden chamber (Nucleopore, Tübingen, Germany) as previously described [9]. For quantification, migrated cells were counted by 20 high-power fields in four different wells using the Axioskop 2 Plus microscope (Carl Zeiss, Jena, Germany).

#### 2.2.5. Immunoprecipitation and Phosphatase Activity Measurement

Immunoprecipitation and the measurement of SHP-2 activity were done as previously described [9,23]. All the steps related to the SHP-2 activity were performed on ice and inside an anoxic chamber to prevent the spontaneous oxidation of the catalytic cysteine of SHP-2 (GB2202-P-V, MBraun, Garching Germany). The activity of SHP-2 was measured in the immunoprecipitates using the Malachite-Green based-Tyrosine Phosphatase Assay System (Promega, Walldorf, Germany). 

#### 2.2.6. Western Blotting

For Western blotting, 5–10 × 10^6^ human monocytes or 5 × 10^6^ THP-1 cells were lysed using RIPA buffer containing freshly added protease and phosphatase inhibitor cocktail (Thermo Scientific). Equal amounts of proteins were run on a 10% SDS-PAGE. Membranes were incubated with SHP-2 primary antibody (mouse, SC-7384) or Vinculin (mouse, SC-73614) from Santa Cruz Biotechnology (SCB), at a 1/1000 dilution in NET g blocking solution overnight at 4 °C. HRP-conjugated mouse IgG binding proteins (SC-525409) were used instead of secondary antibodies. Blots were developed using Pierce ECL substrate (Thermo Scientific, Karlsruhe, Germany), and the images were captured using Amersham Imager 600 (GE Healthcare, Solingen, Germany). Using Fiji software, the band intensity was quantified and normalised to internal control (Vinculin).

#### 2.2.7. ELISA-based Cytokine Measurement

Monocytes were exposed to 300 µM MG with and without SHP-2 inhibitor for 24 h. After that, the supernatant was collected and frozen at −20 °C until analysis. To detect TNFα, IL-6 and IL-1β, the DuoSet ELISA kits from R&D systems, Minneapolis, USA (DY210, DY206 and DY201, respectively) were used according to the manufacturer’s instructions. Absorbance was measured at 450 nm using Victor X3 plate reader (Perkin Elmer, Waltham, MA, USA). Quantification of the TNFα, IL-6 and IL-1β levels were done using Four-Parameter logistic regression method. 

#### 2.2.8. FACS Staining

FACS antibodis were from Abcam (Cambridge, the UK). 1.5 × 10^6^ Monocytes treated with MG for 24 h were washed once with 1× ice-cold PBS and fixed with 80% Methanol. Staining of the surface RAGE or isotype control was carried out in FACS buffer (PBS with 1% BSA and 0.1% sodium azide) for 30 min at 4 °C using an anti-RAGE antibody (Abcam, ab54741 or isotype antibody (Abcam, ab170191). After that, the cells were stained using the Alexa Fluor^®^ 488 conjugated secondary antibody (Abcam, ab150113) for 20 min at 4 °C in the dark. After staining, the cells were washed with 1× PBS, and FACS analysis was performed using Guava easyCyte (Millipore, Darmstadt, Germany).

#### 2.2.9. Electrophoretic Mobility Shift Assays (EMSA)

Nuclear extracts were prepared from monocytes using NE-PER Nuclear and Cytoplasmic Extraction Reagents (Thermo Scientific, Karlsruhe, Germany), and EMSA was performed using a LightShift Chemiluminescent EMSA Kit (Thermo Scientific, Karlsruhe, Germany) according to the manufacturer’s instructions. 8–12 µg of nuclear extracts were pre-incubated for 20 min at room temperature in a 20 μL reaction volume containing 1× binding buffer, 50 ng/μL poly (dI-dC), and 20 pmol of biotin-labelled probes complementary to the NF-κB–binding site in the SHP-2 promoter (5′-GGCTAGGTAATTTCCCTGGTCAT-3′). After that, the complexes were resolved in 6% non-denaturing PAGE gel, and the mobility of biotinylated signals was analysed and captured using Amersham Imager 600 (GE Healthcare, Solingen Germany).

#### 2.2.10. Gene Silencing by siRNA

Monocytes were transfected using the human monocyte nucleofector kit (Lonza, Cologne, Germany) according to the manufacturer’s instructions. 10 × 10^6^ monocytes were used per electroporation reaction. siRNAs were purchased from Santa Cruz Biotechnology, Heidelberg, Germany. The cell pellet resuspended in 100 µL nucleofection solution was mixed with either 50 nM p65 siRNA (Santa Cruz, sc-29410) or control siRNA (Santa Cruz, sc-37007). Nucleofection was carried out in the supplied cuvettes using Program Y-001 in the Nuclefector 2b device (Lonza, Cologne, Gemany). The cells were processed 24 h post-transfection.

#### 2.2.11. RNA Isolation, qPCR and Gene Expression Analysis 

For the extraction of RNA, roughly 5–8 × 10^6^ monocytes were used routinely. For in vitro experiments, the RNA was extracted between 8–12 h post cell treatment. Total RNA purification was performed using NucleoSpin RNA isolation kit (Macherey-Nagel, Dueren, Germany), and cDNA was synthesised using RevertAid First Strand cDNA Synthesis Kit (Thermo Scientific, Karlsruhe, Germany). qPCR was carried out using iTaq™ Universal SYBR^®^ Green supermix (Bio-Rad, Feldkirchen, Germany) in Connect Real-Time PCR Detection System (Bio-Rad, Feldkirchen, Germany). The threshold cycle (Cq) value of each sample was calculated, and the expression of target gene mRNA relative to rplO, Hprt or YWHAZ was determined by the 2−ΔΔCt method. The sequences of the primers used can be found in Supplementary Table S2.

#### 2.2.12. RT-PCR Analysis of RAGE mRNA Splice Variants

Total RNA purification was performed using NucleoSpin RNA isolation kit (Macherey-Nagel, Dueren, Germany), and cDNA was synthesised using RevertAid First Strand cDNA Synthesis Kit (Thermo Scientific, Karlsruhe, Germany). Full-length, C-truncated and N-truncated RAGE splice variants were detected using the primers and PCR conditions as reported previously [24]. The sequences of the primers used can be found in Supplementary Table S3.

#### 2.2.13. Study Design, Blinding and Statistical Analysis 

All the migration experiments were carried out with four technical replicates per condition. *n* = 3–4 were done for all experiments, and specific details about the design of the experiments are given in the figure legends. Animal experiments were blind to group assignment. All statistical analyses are presented as means ± SEM. To analyse significance of differences in experiments with monocytes isolated from diabetic or healthy individuals/mice, the Mann–Whitney Rank Sum Test (for intergroup comparisons) or Kruskal–Wallis One Way Analysis of Variance on Ranks with Tukey or Dunn’s post hoc correction was used. For all the other experiments, two-sample independent *t*-tests or when multiple comparisons were made, Kruskal–Wallis One Way Analysis of Variance on Ranks with Tukey or Dunn’s post hoc correction was performed. SigmaPlot software was used for the statistical analysis. The level of significance was defined as *p* < 0.05. 

#### 2.2.14. Data Availability

The datasets used and/or analysed during the current study are available from the corresponding author on reasonable request.

## 3. Results

### 3.1. T2DM Milieu and AGE Precursor, Methylglyoxal, Induces Upregulation of SHP-2 Expression and Promotes Random Migration of Monocytes

**Figure 1:**
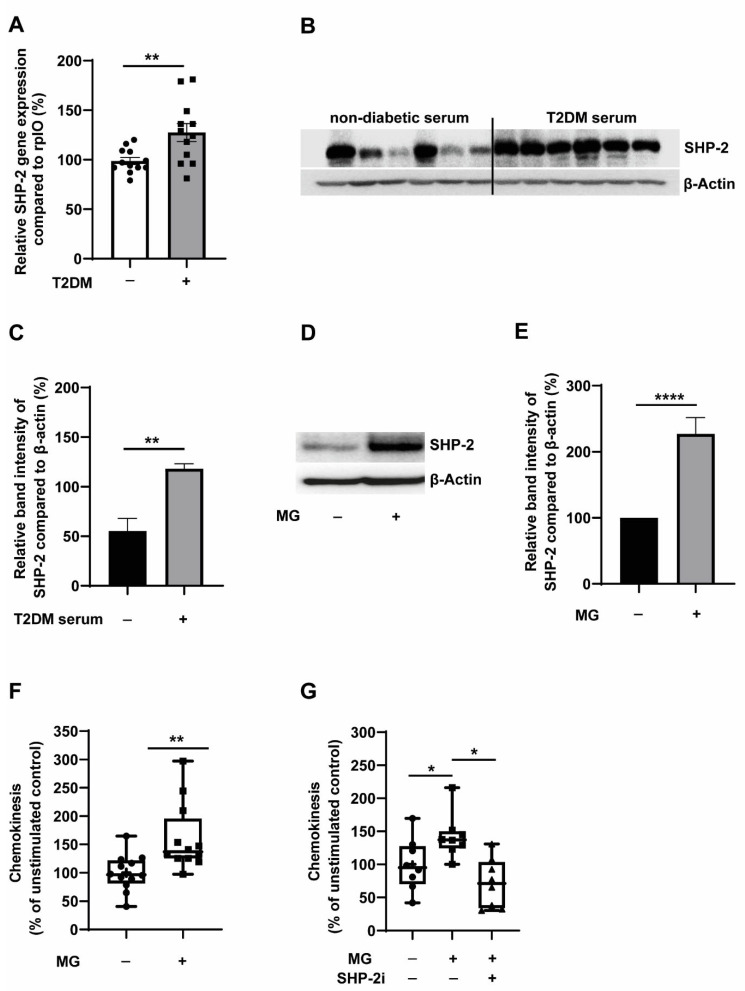
**Hyperglycemia and glucose metabolite-induced upregulation of SHP-2 promotes random migration of monocytes**. (**A**) Relative expression of SHP-2 mRNA compared to rplO. Primary monocytes isolated from the peripheral blood of non-T2DM (*n* = 12) and T2DM (*n* = 12) were used for the analysis. (**B**) Representative blots and (**C**) corresponding quantification. Expression of SHP-2 in relation to ß-actin. Exposure of monocytes with the serum of non-diabetic (black bar) or diabetic patients (grey bar) for 24 h. *n* = 6, respectively. (**D**) Representative blots and (**E**) corresponding quantification. Expression of SHP-2 in relation to ß-actin. Exposure of monocytes without (black bar) or with (grey bar) 300 µM MG for 24 h. *n* = 12. (**F**,**G**) Bars display counted monocytes which migrated completely through the membrane during transwell migration. Circles (Control), squares (exposure with 300 µM MG for 24 hrs) and triangles (exposure with 300 µM MG and treatment with SHP-2i, 700 nM SHP099) represent counted monocytes of each experiment in percentage compared to the mean value of unstimulated control. (**E**) *n* = 12, (**F**) *n* = 8. * *p* < 0,05, ** *p* < 0.01, **** *p* < 0.0001.

We recently identified the role of SHP-2 tyrosine phosphatase in mediating VEGF resistance of T2DM monocytes [9]. To further validate the contribution of T2DM in the regulation of SHP-2 expression, we first analysed the expression of SHP-2 transcripts from the monocytes isolated from T2DM patients and age-and sex-matched healthy individuals. As seen in Figure 1A, we detected a significant elevation of SHP-2 expression in T2DM monocytes. Similarly, elevated expression of murine SHP-2 was detected in monocytes from the leptin receptor-deficient T2DM db/db mice (Supplementary Figure S1A). Sera from T2DM patients contain elevated levels of a plethora of cytokines, growth factors and glucose metabolites [25]. To address if T2DM sera alone could trigger SHP-2 expression upregulation, we cultured non-diabetic monocytes in the presence of serum obtained from T2DM patients. As expected, we detected a robust upregulation of SHP-2 expression in monocytes treated with T2DM sera (Figure 1B,C). The accumulation of glucose metabolite and advanced glycation product (AGE) precursor, methylglyoxal (MG), was recently identified to be a major component of diabetic milieu which was independently able to drive the detrimental effects of hyperglycemia even in the absence of elevated glucose levels [13,26]. Confirming the importance of MG, we readily detected heightened SHP-2 expression when monocytes were treated with MG in normoglycemic conditions (Figure 1D,E).

Furthermore, the upregulation of SHP-2 expression induced by MG resulted in the elevation of SHP-2 phosphatase activity under similar conditions (Supplementary Figure S1B). Since T2DM predisposes individuals to CVDs and monocyte activation—as evident by their enhanced random migration potential (also termed chemokinesis)—is associated with coronary artery disease (CAD) [3], we next analysed the chemokinesis behaviour of monocytes upon MG treatment. Higher random motility of MG-treated monocytes was readily observed, indicating higher monocyte activation (Figure 1F and Figure S1C). To understand the role of SHP-2 tyrosine phosphatase in mediating chemokinesis of MG-monocytes, we used a highly selective small-molecule allosteric SHP-2 inhibitor, SHP099 (mentioned as SHP-2i in the manuscript), that stabilises SHP-2 in an auto-inhibited conformation [27]. Inhibition of SHP-2 using SHP099 completely restored the aberrant pro-migratory phenotype induced by MG, indicating that SHP-2 contributes to monocyte activation in the diabetic milieu (Figure 1G and Figure S1D). To further validate the relevance of SHP-2 in mediating monocyte activation, we employed another small-molecule non-allosteric inhibitor of SHP-2, NSC-87877 [28]. Similar to SHP099, SHP-2 inhibition using NSC-878777 (mentioned as SHP-2i 2 in the manuscript) could completely block the monocyte activation induced by MG (Supplementary Figure S1E,F).

Given the already reported concept of hyperglycemic memory in vascular cells [29], we wondered whether monocytes memorize T2DM-induced SHP-2 expression upregulation and SHP-2-dependent monocyte activation even after the hyperglycemic conditions are reversed. To test this, we used THP-1 monocytic cells and exposed these cells to a diabetic milieu (MG+AGE mimetic) for 24 h, and the expression levels of SHP-2 and monocyte activation status were analysed. Thereafter, the cells rested for 6 days without the diabetic milieu (termed as former hyperglycemic), and then the expression levels of SHP-2 and monocyte activation status were analysed again (Supplementary Figure S2A). As shown in Supplementary Figure S2B–E, and consistent with what was observed in primary monocytes, treatment of THP-1 cells with a diabetic milieu induced monocyte activation resulting in enhanced random motility. We also detected elevated SHP-2 expression in these cells. Interestingly, after 6 days of rest, the former hyperglycemic cells neither displayed elevated random migration phenotype nor showed an expression upregulation of SHP-2 (Supplementary Figure S2F–I). These data demonstrated that SHP-2 expression upregulation induced by the diabetic milieu is entirely reversible. 

### 3.2. SHP-2 Expression Is Linked to Inflammation and Is Regulated by NFκB in Monocytes

**Figure 2:**
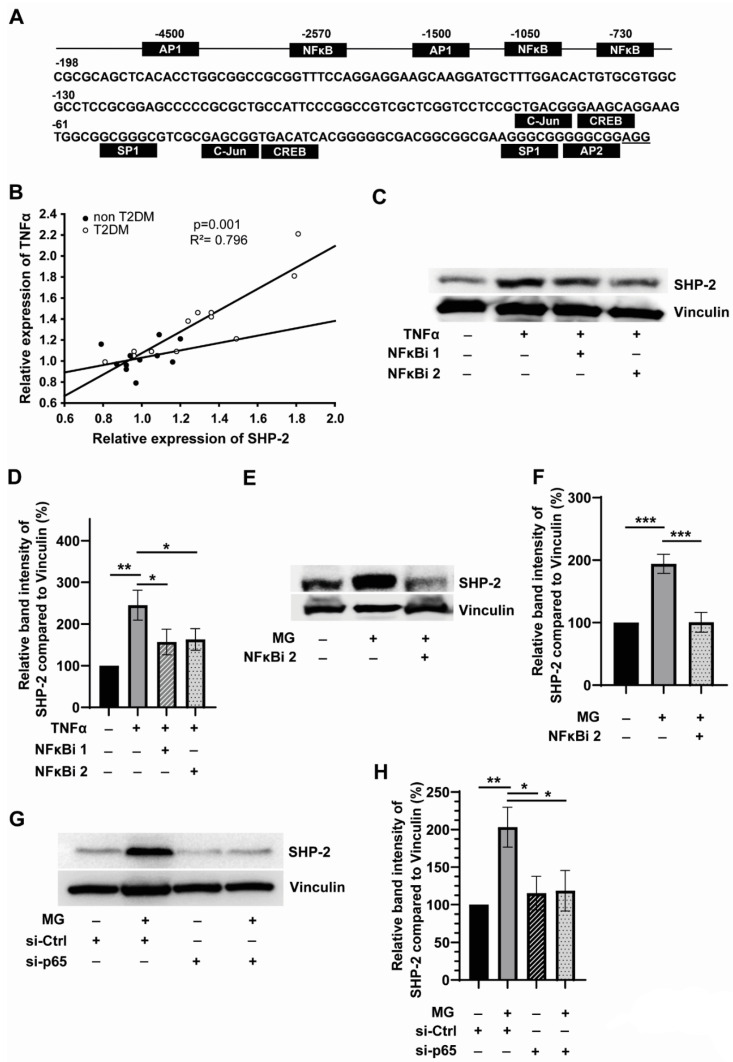
**SHP-2 expression is linked to inflammation and is regulated by NFkB in monocytes.** (**A**) The promoter region of the PTPN11 (SHP-2) gene depicting the binding sites of various transcription factors, including the three NFkB binding sites (**B**) Linear regression analysis of SHP-2 and TNFα transcript levels in non-T2DM (*n* = 12) and T2DM (*n* = 12). (**C**) Representative blots and (**D**) corresponding quantification. Expression of SHP-2 in relation to Vinculin. Exposure of monocytes without (black bar) or with (grey bars) 10 µg/mL TNFa. Quantification of monocytes additionally treated with NFkB-inhibitor is shown as striped (1 µM NF-kB Activation Inhibitor I, NFκBi 1, Calbiochem) and dotted bar (5 µM Activation Inhibitor II, JSH23, NFκBi 2, Calbiochem). *n* = 6. (**E**) Representative blots and (**F**) corresponding quantification. Expression of SHP-2 in relation to Vinculin. Exposure of monocytes without (black bar) or with (grey bars) 300 µM MG for 24 h. Quantification of monocytes additionally treated with NFkB-inhibitor is shown as the dotted bar (5 µM Activation Inhibitor II, JSH23, NFκBi 2). *n* = 6. (**G**) Representative blots and (**H**) corresponding quantification. Expression of SHP-2 in relation to Vinculin. Exposure of Monocytes without (black bar) or with (grey bars) 300 µM MG for 24 h. Striped and dotted bars indicate monocytes which were additionally transfected with siRNA against NFkB-p65. *n* = 5. * *p* < 0,05, ** *p* < 0.01, *** *p* < 0.001.

Since aberrant expression and activity of SHP-2 phosphatase drives monocyte activation, we wondered how SHP-2 gene expression is regulated in diabetic monocytes. To understand the potential transcriptional regulation of SHP-2, we first analysed the promoter region of SHP-2/PTPN11 gene. PTPN11 promoter has no consensus TATA sequence for RNA polymerase-II-initiated transcriptional activation. However, a binding motif for Sp1 transcription factor was present upstream of the predicted transcription start site indicating that Sp1 regulates the basal expression of PTPN11 (Figure 2A). Notably, three NFκB consensus binding motifs were present in the PTPN11 promoter region (Figure 2A), indicating that activation of NFκB-dependent inflammatory signalling pathways drives PTPN11 gene expression. TNFα-induced, NFκB-dependent inflammatory responses has been implicated in the pathogenesis of T2DM [30]. We, therefore, analysed whether there is any correlation between TNFα and SHP-2 expression in T2DM monocytes.

Interestingly, linear regression analysis revealed elevated TNFα levels correlated significantly with SHP-2 expression levels in monocytes isolated from T2DM patients (Figure 2B). To further confirm the role of TNFα-NFκB axis in regulating SHP-2 expression, we treated monocytes with exogenous TNFα in the presence or absence of two different NFκB inhibitors, NFκBi 1 (quinazoline-based inhibitor of NFκB transcriptional activation) and NFκBi 2 (JSH23; selective inhibitor of nuclear translocation of p65 subunit). The blockade of NFκB activation readily led to the reversal of TNFα-induced SHP-2 expression (Figure 2C,D), validating that SHP-2 is indeed an NFκB target gene in monocytes. Furthermore, co-treatment with both NFκB inhibitors slightly improved the TNFα-induced SHP-2 expression (Supplementary Figure S3A,B). As shown in Figure 1D in the previous results section, the glucose metabolite and advanced glycation product (AGE) precursor, methylglyoxal (MG), contributes to the upregulation of SHP-2 phosphatase. Therefore, we wondered whether MG induces SHP-2 expression by activating the NFκB pathway. As expected, co-application of either of the two different NFκB inhibitors significantly reversed the MG-induced SHP-2 expression (Figure 2E,F and Figure S4A,B). Furthermore, co-treatment with both NFκB inhibitors slightly improved the MG-induced SHP-2 expression (Supplementary Figure S3C,D). In order to further validate the role of NFκB in mediating MG-induced SHP-2 expression, we knocked down the p65 subunit of the NFκB using siRNA. Even with a knockdown efficiency of 50% (Supplementary Figure S4C,D), we could significantly abrogate the ability of MG to induce SHP-2 expression (Figure 2G,H). This data further confirmed that MG promotes inflammation induction in monocytes, and MG-induced NFκB drives SHP-2 expression. Next, we explored whether SHP-2 has any role in mediating MG-induced inflammation through a positive feedback mechanism. In line with the results that MG induces NFκB, we detected elevated levels of secreted IL6 and TNFα and a slightly elevated IL-1β (non-significant) in monocytes exposed to MG. However, pharmacological inhibition of SHP-2 using SHP099 did not influence MG-induced pro-inflammatory cytokine release (Supplementary Figure S4E–G), indicating that SHP-2 does not play an active role in inducing inflammation mediated by MG. 

### 3.3. T2DM Milieu Induces RAGE Expression in Monocytes, and AGE-RAGE Signalling Potentiates SHP-2 Expression through the NFκB Pathway

**Figure 3:**
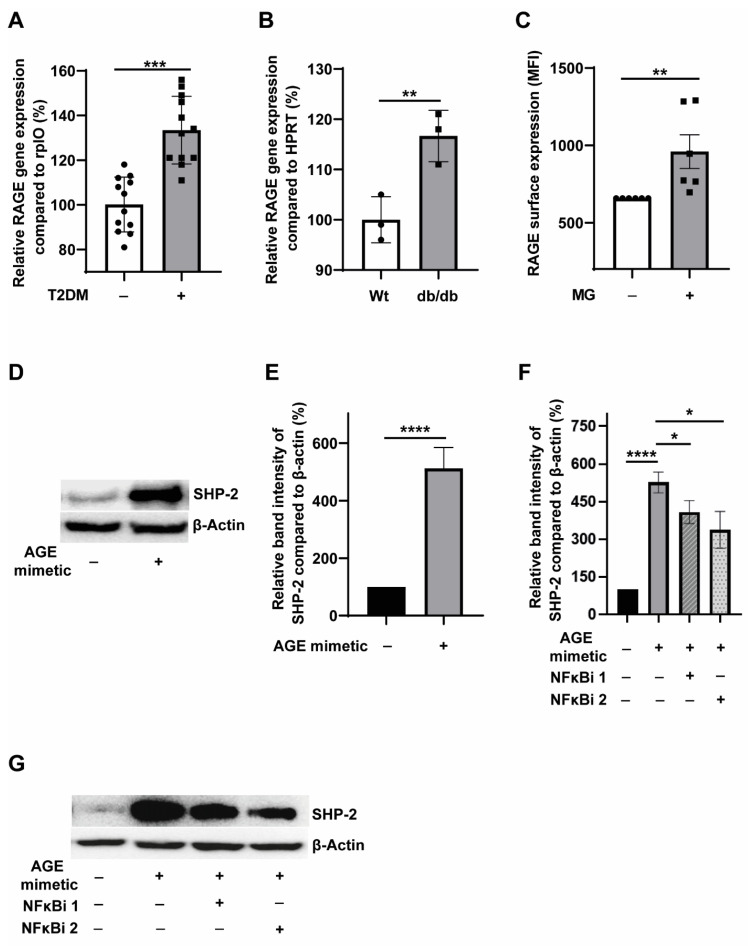
**Hyperglycemia induces RAGE expression in monocytes and AGE-RAGE signaling potentiates SHP-2 expression through the NFkB pathway**. (**A**) Relative expression of RAGE mRNA compared to rplO. Primary monocytes isolated from the peripheral blood of non-T2DM (*n* = 12) and T2DM (*n* = 12) were used for the analysis. (**B**) Relative expression of RAGE mRNA compared to HRPT. Primary murine monocytes isolated from the bone marrow of wildtype (*n* = 3) and db/db (*n* = 3) mice were used for the analysis. (**C**) FACS analysis of the surface expression of RAGE. Exposure of monocytes with 300 µM MG for 24 h. *n* = 6. (**D**) Representative blots and (**E**) corresponding quantification. Expression of SHP-2 in relation to ß-actin. Exposure of monocytes without (black bar) or with (grey bar) 150 µg/mL AGE-BSA for 24 h. *n* = 11. (**F**) Corresponding quantification and (**G**) representative blots. Expression of SHP-2 in relation to ß-actin. Exposure of monocytes without (black bar) or with (grey bars) 150 µg/mL AGE-BSA for 24 h. Quantification of monocytes additionally treated with NFkB-inhibitor is shown as striped (5 µM Activation Inhibitor II, NFκBi 2, JSH23) and dotted bar (1 µM NF-kB Activation Inhibitor, NFκBi 1). *n* = 6. * *p* < 0,05, ** *p* < 0.01, *** *p* < 0.001, **** *p* < 0.0001.

Methylglyoxal is an AGE precursor, and the interactions of AGE with its cognate receptor RAGE lead to inflammation induction [31]. Moreover, RAGE signalling is pivotal for developing atherosclerosis in the diabetic background [32], and elevated levels of RAGE have been detected in the atherosclerotic plaques of diabetic subjects [33]. However, the expression levels of RAGE in T2DM monocytes remain obscure. In order to understand the expression of RAGE levels in T2DM, we first analysed the RAGE transcript levels in the monocytes obtained from T2DM patients and non-T2DM controls. As shown in Figure 3A, we detected significantly elevated RAGE mRNA levels in T2DM monocytes. Likewise, we also detected elevated transcripts of RAGE in the monocytes from the leptin receptor-deficient T2DM db/db mice (Figure 3B). Taken together, these data confirmed that the T2DM milieu potentiates the expression of AGE receptor, RAGE, in monocytes. We then asked whether MG treatment could enhance RAGE expression in monocytes. FACS analysis revealed elevated surface expression of RAGE on monocytes treated with MG (Figure 3C).

Furthermore, AGE mimetic treatment also induced the surface expression of RAGE in monocytes (Supplementary Figure S5A). S100B is a well-established RAGE ligand [34]. Both AGE mimetic, AGE-BSA and S100B induced the expression of RAGE mRNA in monocytes (Supplementary Figure S5B). These data indicate that the activation of AGE-RAGE signalling promotes RAGE expression. We also detected an upregulation of RAGE transcripts in monocytic THP-1 cells when treated with prolonged hyperglycemic conditions for five days (Supplementary Figure S5C). The most likely explanation for this finding is that the accumulation of glycation end products to stimulate RAGE expression is gradual, whereas when cells are treated directly with AGE precursors and AGE mimetics, RAGE expression induction is robust. Since it is known that RAGE has several splice variants [35], we also investigated whether potentiation of the expression of any particular splice variant of RAGE occurs during hyperglycemic conditions. Monocytes and THP-1 monocytic cells expressed full RAGE and C-truncated but not N-truncated RAGE splice variants (Supplementary Figure S5D). Furthermore, we could not detect any alterations in the levels of full RAGE and C-truncated isoforms upon hyperglycemic conditions (Supplementary Figure S5D).

To address whether SHP-2 expression could be stimulated by activating the AGE-RAGE signalling axis, we used AGE mimetic AGE-BSA (glycated BSA). AGE-BSA is a commonly used AGE-mimetic to induce AGE-RAGE signalling [36]. Treatment of monocytes with the AGE-mimetic robustly induced SHP-2 expression (Figure 3D,E). Next, we asked whether NFκB drives the AGE-RAGE signalling mediated SHP-2 upregulation. Indeed, blockade of NFκB by two different pharmacological inhibitors significantly reduced AGE mimetic induced SHP-2 expression (Figure 3F,G). Furthermore, co-treatment with both NFκB inhibitors slightly improved the AGE mimetic-induced SHP-2 expression (Supplementary Figure S3A,B). These results further prove that NFκB tightly regulates SHP-2 expression in primary monocytes. 

### 3.4. Pharmacological Targeting of RAGE Reverses MG-Induced SHP-2 Expression and Activity through the Inhibition of NFκB-p65 Binding to the SHP-2 Promoter

**Figure 4:**
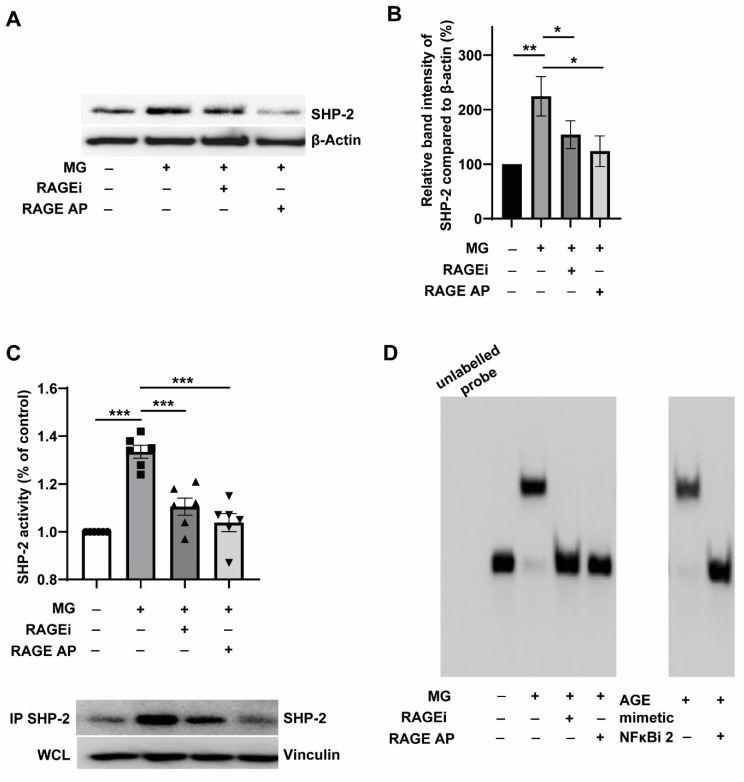
Genetic and pharmacological targeting of RAGE reverses MG-induced SHP-2 expression and activity through the inhibition of NFkB binding to the SHP-2 promoter. (**A**) Representative blots and (**B**) corresponding quantification. Expression of SHP-2 in relation to ß-actin. Exposure of monocytes without (black bar) or with (grey bars) 300 µM MG for 24 h. Quantification of monocytes additionally treated with RAGE-inhibitor (RAGEi, 200 nM FPS-ZM1, Calbiochem) is shown as striped or with RAGE-antagonist peptide (RAGE AP, 2 µM, Calbiochem) as the dotted bar. *n* = 8. (**C**) SHP-2 phosphatase activity assay. Monocytes were treated with 300 µM MG for 24 h with and without RAGE-inhibitor (RAGEi, 200 nM FPS-ZM1), or with RAGE-antagonist peptide (RAGE AP, 2 µM). Thereafter, the cells were lysed in anoxic conditions and the phosphatase activity was measured in the SHP-2 immunoprecipitates using the Tyrosine Phosphatase Assay System (Promega). The SHP-2 levels in the immunoprecipitates were detected using Western Blot (lower panel). *n* = 6. (**D**) Electrophoretic Mobility Shift Assay (EMSA) assay. Monocytes were exposed to 300 µM MG or 150 µg/mL AGE-BSA for 24 h in the presence or the absence of RAGE-inhibitor (RAGEi, 200 nM FPS-ZM1), RAGE-antagonist peptide (RAGE AP, 2 µM) or 5 µM NFκB Activation Inhibitor II, NFκBi 2, JSH23). Thereafter, the nuclear extracts were prepared, and the EMSA was carried out using LightShift Chemiluminescent EMSA Kit (Thermo Scientific), *n* = 3. * *p* < 0,05, ** *p* < 0.01, *** *p* < 0.001.

It was clear from the results described so far that inflammatory stimuli like TNFα drive SHP-2 expression, and AGE precursor, MG, and AGE mimetic AGE-BSA, could also stimulate SHP-2 expression upregulation. All these stimulants upregulated SHP-2 through NFκB activation. From Figure 3C, it was evident that MG can upregulate RAGE expression. However, it was not clear whether MG-induced SHP-2 expression in monocytes is RAGE receptor-dependent or not. To address this question, we used two different approaches to inhibit RAGE. FPS-ZM1 (described as RAGEi) is a recently described multimodal RAGE-specific inhibitor that could inhibit RAGE-mediated NFκB activation [37]. RAGE antagonist peptide (described as RAGE AP) is also described as a potent blocker of AGE-RAGE activation of NFκB [38]. As shown in Figure 4A,B, co-treatment of monocytes with RAGEi or RAGE AP significantly reduced MG-induced SHP-2 expression. The effect of RAGE AP in blocking the MG-induced SHP-2 expression seemed to be more pronounced than FPS-ZM1 (Figure 4A,B). However, the ability of both RAGE antagonists to block MG-induced SHP-2 expression confirmed that MG primarily transduces its effects on SHP-2 expression through the RAGE receptor. Under similar conditions, RAGEi and RAGE AP suppressed the SHP-2 mRNA levels (Supplementary Figure S6A), confirming that RAGE signalling drives MG-induced SHP-2 transcription. Consistent with the pivotal role of RAGE on MG-induced SHP-2 expression, blockade of RAGE suppressed MG-induced SHP-2 tyrosine phosphatase activity (Figure 4C). In addition, the stimulation of SHP-2 phosphatase activity by the AGE mimetic treatment could be entirely reversed by NFκB inhibition (Supplementary Figure S6B). The SHP-2 activity data indicated that MG/AGE mimetic controls SHP-2 expression through the RAGE-NFκB signalling axis.

To further confirm that MG-induced effects on SHP-2 expression are through the RAGE-dependent binding of NFκB-p65 to the SHP-2 promoter, we carried out Electrophoretic Mobility Shift Assays (EMSA) using nuclear extracts. As shown in Figure 4D, both MG and AGE mimetic treatment resulted in the shifted band, indicating the binding of NFκB-p65 to the SHP-2 promoter. This mobility shift, induced by MG, was abolished entirely when cells were co-treated with RAGEi and RAP. This indicated that MG-induced binding of NFκB-p65 to the SHP-2 promoter is mediated by RAGE signalling. Furthermore, abolition of the mobility shift induced by AGE mimetic treatment by a selective inhibitor of nuclear translocation of p65 subunit (NFκBi 2; JSH23) indicated that AGE imparts its effect on SHP-2 expression by promoting the binding of NFκB-p65 to the SHP-2 promoter (Figure 4D).

### 3.5. RAGE Ligation Promotes, and RAGE Inhibition Attenuates the Random Motility of Monocytes

**Figure 5:**
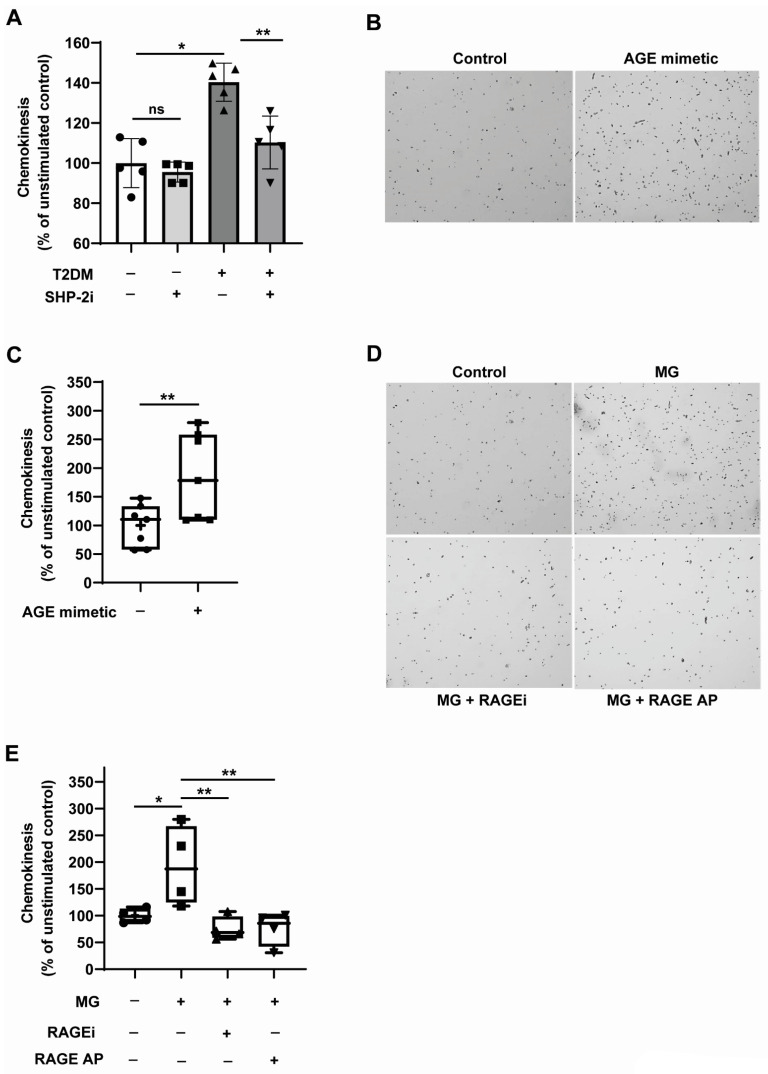
**RAGE ligation accelerates and RAGE inhibition attenuates the MG-induced random motility of monocytes.** (**A**) Random motility analysis of monocytes from T2DM patients. Primary monocytes isolated from the peripheral blood of non-T2DM (*n* = 5) and T2DM (*n* = 5) were treated with 400 nM of the SHP-2i (NSC 878777) for 8 h. After that, the monocytes were analysed for their migratory potential using Boyden chamber assays. (**B**) Representative pictures of automatically counted monocytes that migrated completely through the membrane during transwell migration and (**C**) corresponding quantification. Circles (Control) and squares (exposure with 150 µg/mL AGE-BSA for 24 h) represent counted monocytes of each experiment in percentage compared to the mean value of unstimulated control. *n* = 7. (**D**) Representative pictures of automatically counted monocytes which migrated completely through the membrane during transwell migration and (**E**) corresponding quantification. Circles (Control) represent untreated counted monocytes of each experiment in percentage compared to the mean value of unstimulated control. Other samples were treated with 300 µM MG for 24 h. Squares indicate monocytes with functional RAGE. Triangles show monocytes treated either with RAGE-inhibitor (aligned upwards) or RAGE antagonist peptide (aligned downwards). *n* = 4. * *p* < 0.05, ** *p* < 0.01.

Monocyte activation and subsequently enhanced transmigration across the vessel wall in hypercholesterolenemic and T2DM conditions is a predominant event in the development of atherosclerosis [1]. Accelerated random migration of monocytes, resulting from its activation, potentially contributes to the enhanced recruitment of monocytes. From the results described in the previous sections, it is clear that SHP-2 gets upregulated in T2DM conditions. To understand the potential role of the upregulated SHP-2 in mediating the activation of T2DM monocytes, we pharmacologically inhibited SHP-2 and analysed the chemokinesis of monocytes as the readout of monocyte activation. As expected, pharmacological inhibition of SHP-2 significantly reversed the enhanced chemokinesis exhibited by T2DM monocytes (Figure 5A). This data, along with the data shown in Figure 1F, indicated clearly that the elevated SHP-2 expression induced by T2DM milieu directly mediates monocyte activation. In order to understand the contribution of RAGE signalling in mediating monocyte chemokinesis, we exposed monocytes to AGE mimetic, AGE-BSA. AGE-BSA robustly induced the random motility of monocytes (Figure 5B,C). Since we identified that MG induces monocyte activation through SHP-2 (Figure 1F,G) and MG-induced SHP-2 expression requires RAGE (Figure 4A,B), we questioned whether pharmacological targeting of the RAGE pathway is sufficient to block MG-induced monocyte activation. To address this question, the chemokinesis behaviour of MG-treated monocytes was analysed upon the pharmacological inhibition of RAGE. Figure 5D,E show that MG-induced monocyte chemokinesis was reversed entirely when RAGE was inhibited using RAGEi and RAGE AP. Taken together, these data demonstrate that RAGE signalling activation is responsible for the SHP-2 mediated monocyte activation in the T2DM environment and that the blockade of RAGE is sufficient to attenuate SHP-2-dependent monocyte activation. 

## 4. Discussion

Targeting the activation and transmigration of monocytes in various cardiovascular risk factor conditions is a potent approach to alleviate CVD development. Indeed, monocyte recruitment is an indispensable event for atherosclerotic plaque progression [39]. Various approaches to attenuate monocyte recruitment as a strategy to reduce atherosclerosis were investigated in the past. These include targeting MCP-1 [40], MCP-1 receptor CCR2 [41], RANTES-CCR5 axis [42] and Macrophage migration inhibitory factor (MIF) [43]. SHP-2 regulates monocyte activation [9], and consistent with this finding, pharmacological targeting of SHP-2 tyrosine phosphatase was recently reported to be anti-atherosclerotic by regulating VSMC proliferation [20]. Blocking the function of SHP-2 could potentially reduce monocyte activation; however, as SHP-2 is involved in regulating various physiological functions, it was essential to identify the upstream regulators that control SHP-2 expression upregulation in T2DM conditions. We report a hitherto unknown signalling axis regulating SHP-2 expression in T2DM monocytes. The RAGE-NFκB axis precisely controls SHP-2 expression in T2DM conditions, and the pharmacological and genetic targeting of this upstream signalling axis was sufficient to blunt SHP-2 expression and attenuate SHP-2-induced monocyte activation. 

Our analysis revealed the importance of RAGE in inducing monocyte activation through SHP-2 induction. Indeed, RAGE plays a vital role in developing various diabetes-associated disease conditions and is known to be enriched in the diabetic vasculature [44]. Furthermore, the relevance of RAGE in contributing to atherosclerosis development is well characterised [45,46]. Typically, RAGE signalling activation potentiates inflammation through the activation of NFκB and most of the beneficial effects of RAGE blockade in alleviating atherosclerosis development are through the reduction of vascular inflammation [46]. Our results are also consistent with the notion that RAGE activation drives inflammation. In addition, we identified that RAGE signalling activation directly potentiates the expression upregulation of SHP-2 phosphatase through inflammation induction. Activation of SHP-2 then switches on a myriad of signalling alterations leading to monocyte activation. Even though we did not detect any direct connection between SHP-2 expression and inflammation in our experimental system, SHP-2 contributes to macrophage inflammation by attenuating IL-10 signalling [47] and endothelial inflammation through the regulation of NO production [48]. Therefore, the existence of a positive feedback loop where SHP-2 contributes to the potentiation of inflammation under T2DM conditions in vivo remains a possibility and requires further investigation.

Our data also reveal the importance of the glucose metabolite, methylglyoxal, in the induction of the RAGE-NFκB signalling axis, which switches on SHP-2 and drives monocyte activation. Therefore, MG plays a vital role in initiating this detrimental signalling cascade. The notion that MG is an independent driver of vascular complications associated with T2DM is recently gaining momentum. Elevated MG alone is sufficient to drive insulin resistance, obesity and hyperglycemia [13]. Consistent with this finding, we also report that MG is the primary driver of monocyte activation in our experimental system. MG-induced RAGE expression upregulation in monocytes is the primary event that leads to monocyte activation. 

**Figure 6:**
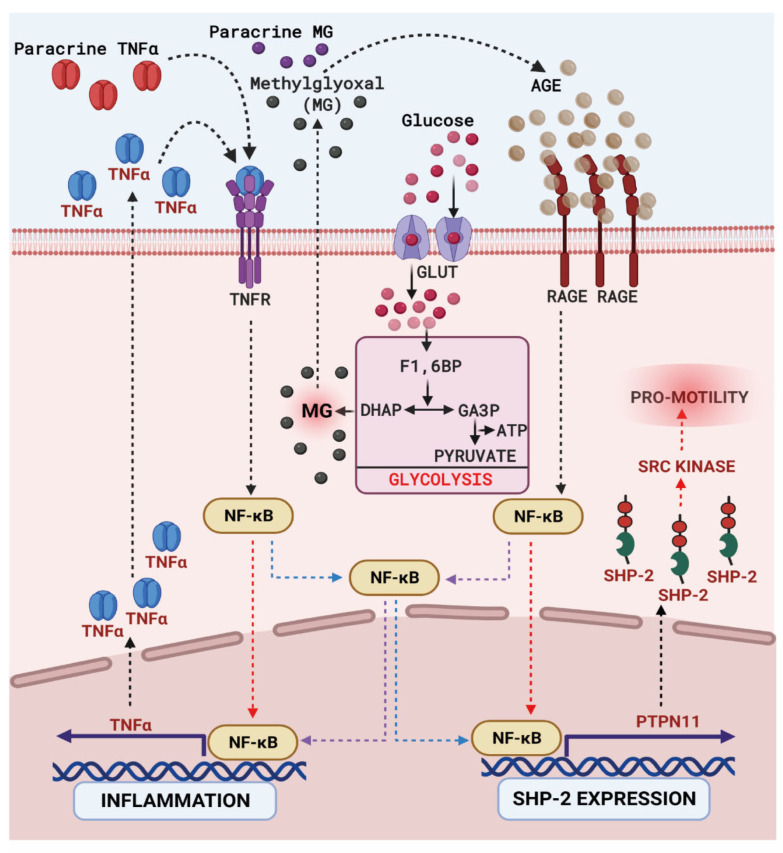
**Model depicting the role of RAGE-NFkB signalling axis in regulating SHP-2 expression in hyperglycemic monocytes.** In T2DM patients, enhanced intracellular glucose transport leads to an elevated glycolysis rate. This results in the accumulation of a highly reactive glucose metabolite, methylglyoxal (MG). MG leads to the formation of advanced glycated end products (AGEs), which can bind to the Receptor for AGEs (RAGE) on the cell surface of monocytes. MG generated in a paracrine manner could also induce the RAGE signalling. Upon the induction of RAGE signalling, transcription factor NFkB gets activated and translocated into the cell nucleus. There, it binds to the promoter of SHP-2, provoking the enhanced transcription of the tyrosine phosphatase. Simultaneously, the RAGE-induced NFκB promotes the production of TNFα. TNFα stimulates the TNFα receptor (TNFR) on the surface of monocytes in an autocrine or paracrine fashion. Activation of TNF signalling accelerates nuclear NFκB accumulation, leading to SHP-2 expression upregulation. Both these events are mutually exclusive. Subsequently, the elevated expression of SHP-2 induces monocyte activation by provoking a pro-migratory phenotype in monocytes.

Identification of the upstream mediators of SHP-2 expression in monocytes is the primary focus of this study. As shown in Figure 6, there is a possibility of significant crosstalk between different vascular cells to initiate several signalling cascades. Methylglyoxal, AGE and TNFα could act in an autocrine or paracrine fashion. Two pathways are operating simultaneously, which drive SHP-2 expression. These are Glucose-MG-AGE-RAGE-NFκB and TNFα-NFκB pathways. Clearly, both pathways are not mutually exclusive. It is important to note that even in the absence of T2DM, low-grade inflammation induced by the hypercholesterolemic (HC) conditions could stimulate the TNFα-NFκB pathway leading to SHP-2 expression and monocyte activation. Intriguingly, there are also reports of the involvement of AGE-RAGE axis in HC conditions [49]. Therefore, it is imperative to assume that the AGE-RAGE-NFκB pathway contributes to monocyte activation in T2DM and HC conditions. However, the complexity at which vascular dysfunction is induced by T2DM is enormous. There is a potent contribution from the polyol pathway to induce vascular cell dysfunction in T2DM conditions. The aldose reductase enzyme transduces several alterations leading to vascular complications independent of RAGE or NFκB [50]. Even though some of the actions of the polyol pathway converge at RAGE [51], several maladaptive responses induced by the activation of polyol pathway in diabetes may not get inhibited if RAGE or NFκB signalling is blocked. Therefore, the potential involvement of the polyol pathway in the regulation of monocyte activation requires further investigation.

In summary, we identified that the blockade of the RAGE-NFκB-SHP-2 axis circumvents monocyte activation in T2DM conditions. Indeed, the blockade of RAGE is attractive due to its lack of any physiological function compared to NFκB and SHP-2. However, further work will be required to establish the relevance of this strategy as a viable therapeutic approach to attenuate monocyte activation in the T2DM environment.

## Data Availability

Not applicable.

## Supplementary Materials

The following supplemtary information can be downloaded at: https://www.mdpi.com/article/10.3390/cells12030513/s1, Figure S1: SHP-2 controls the random migration of monocytes; Figure S2: Diabetic milieu-induced SHP-2 expression and THP-1 monocytic cell activation are reversible; Figure S3: Dual inhibition of NFκB attenuates MG-, AGE-mimetic- and TNFα-induced SHP-2 expression; Figure S4: MG-induced SHP-2 expression is driven by NFκB but it does not contribute to MG-induced inflammation induction; Figure S5: Expression of RAGE in monocytes and its splice variants; Figure S6: The RAGE-NFκB axis controls SHP-2 expression activity; Table S1: Clinical characteristics of the non-T2DM and T2DM individuals used in the study; Table S2: RT-qPCR primer sequences used in the study; Table S3: Splice variant detection of RAGE: RT-PCR primers used.
