# Supplementary material for: Pharmacological Targeting of the RAGE-NFκB Signalling Axis Impedes Monocyte Activation under Diabetic Conditions through the Repression of SHP-2 Tyrosine Phosphatase Function
Source: Cells. 2023 Feb 3;12(3):513. doi: 10.3390/cells12030513 (PMC9914555; doi:10.3390/cells12030513)
Supplement: Supplementary file 1 [file cells-12-00513-s001.zip › cells-1871709-supplementary.pdf]

Supplementary Material

1 Supplementary Figures and Tables

1.1 Supplementary Figures

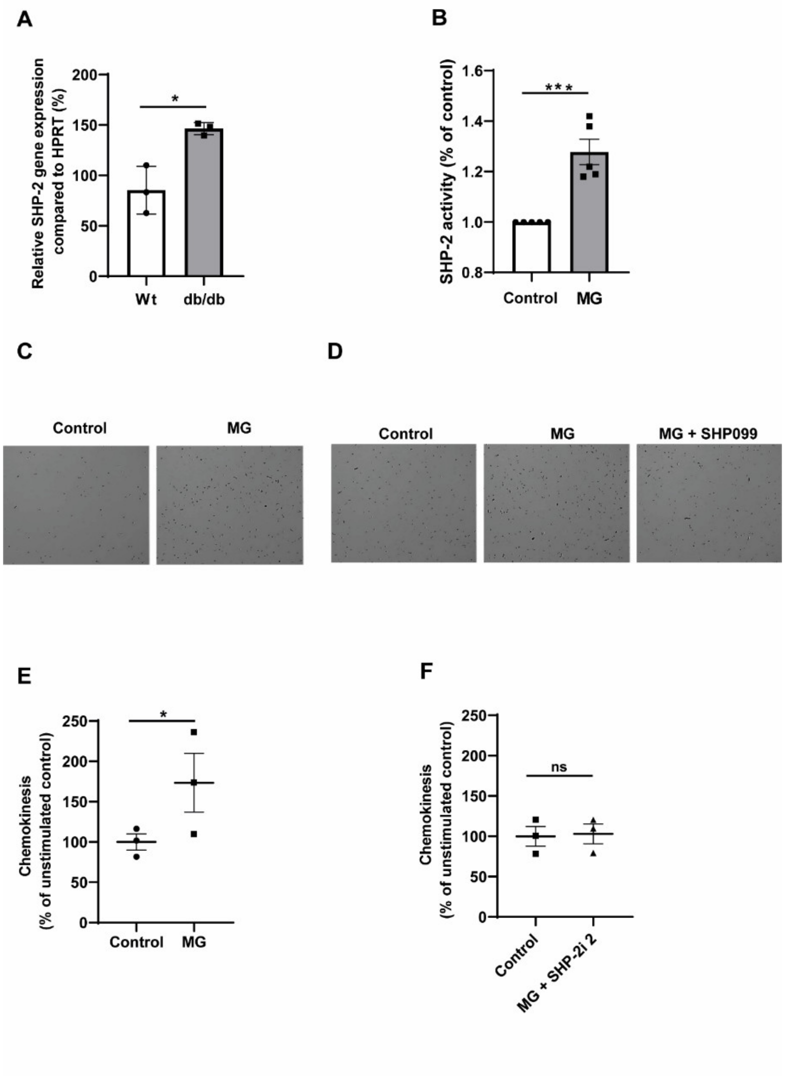

SUPPLEMENTARY FIGURE 1

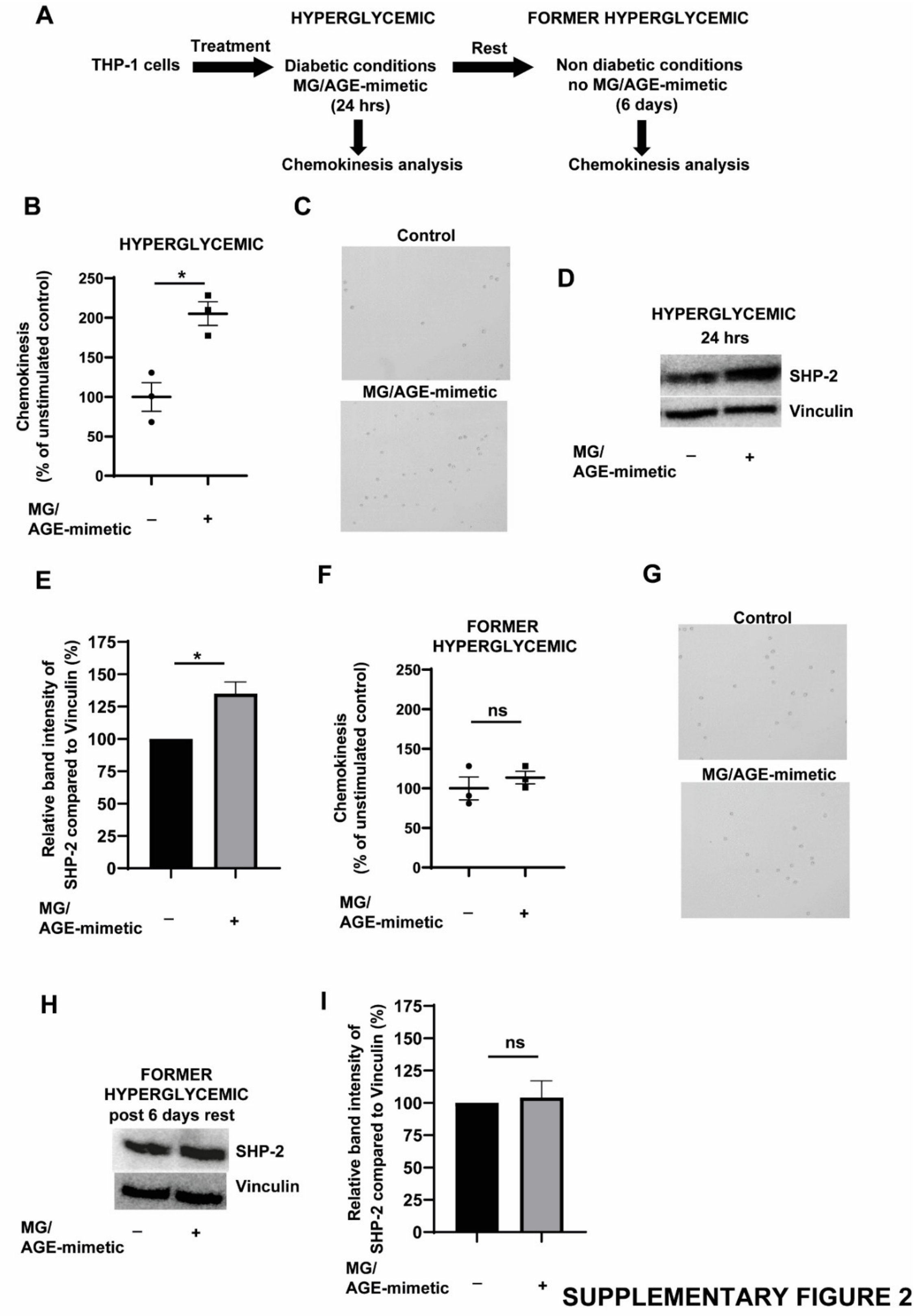

SUPPLEMENTARY FIGURE 2

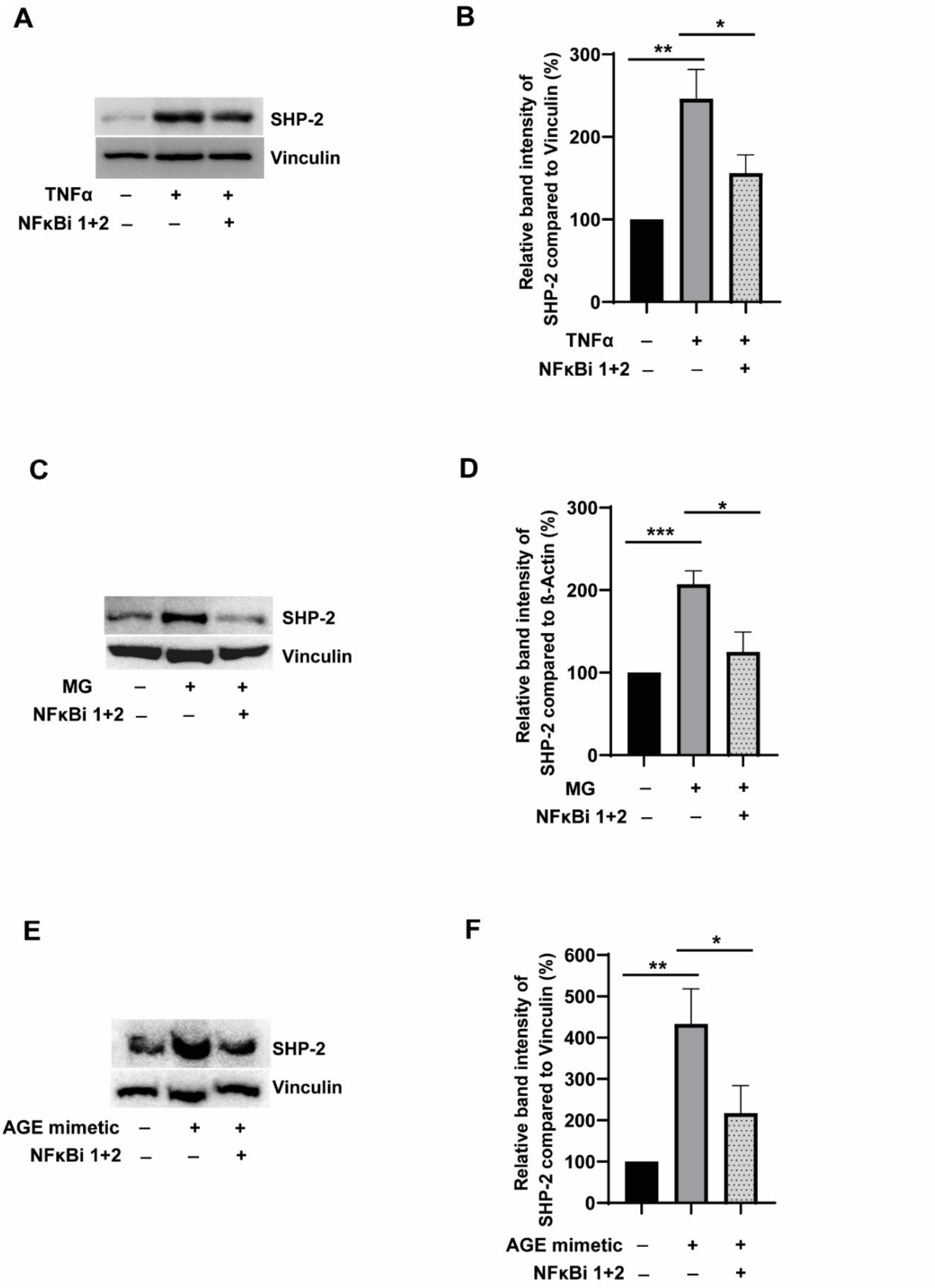

SUPPLEMENTARY FIGURE 3

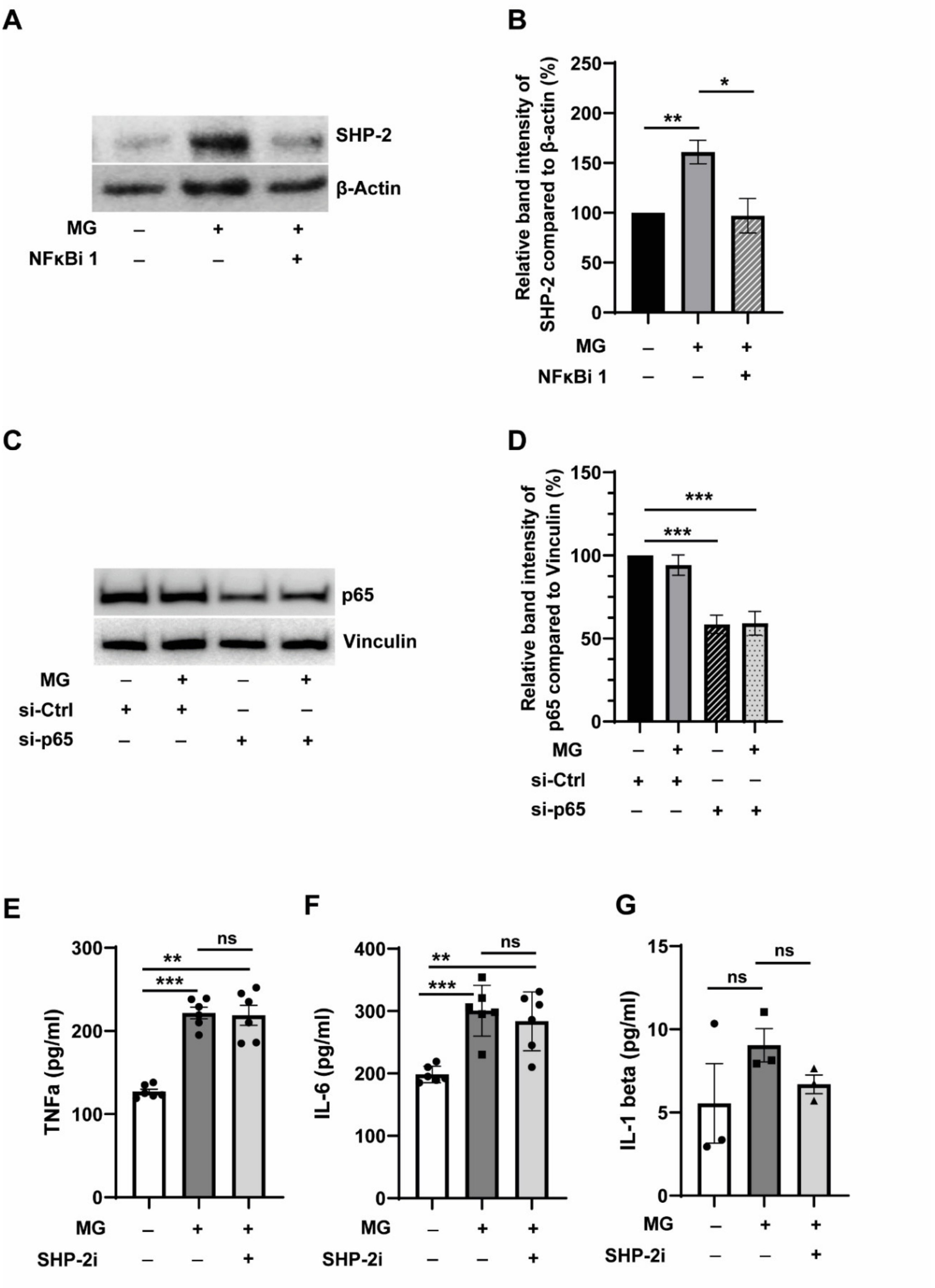

SUPPLEMENTARY FIGURE 4

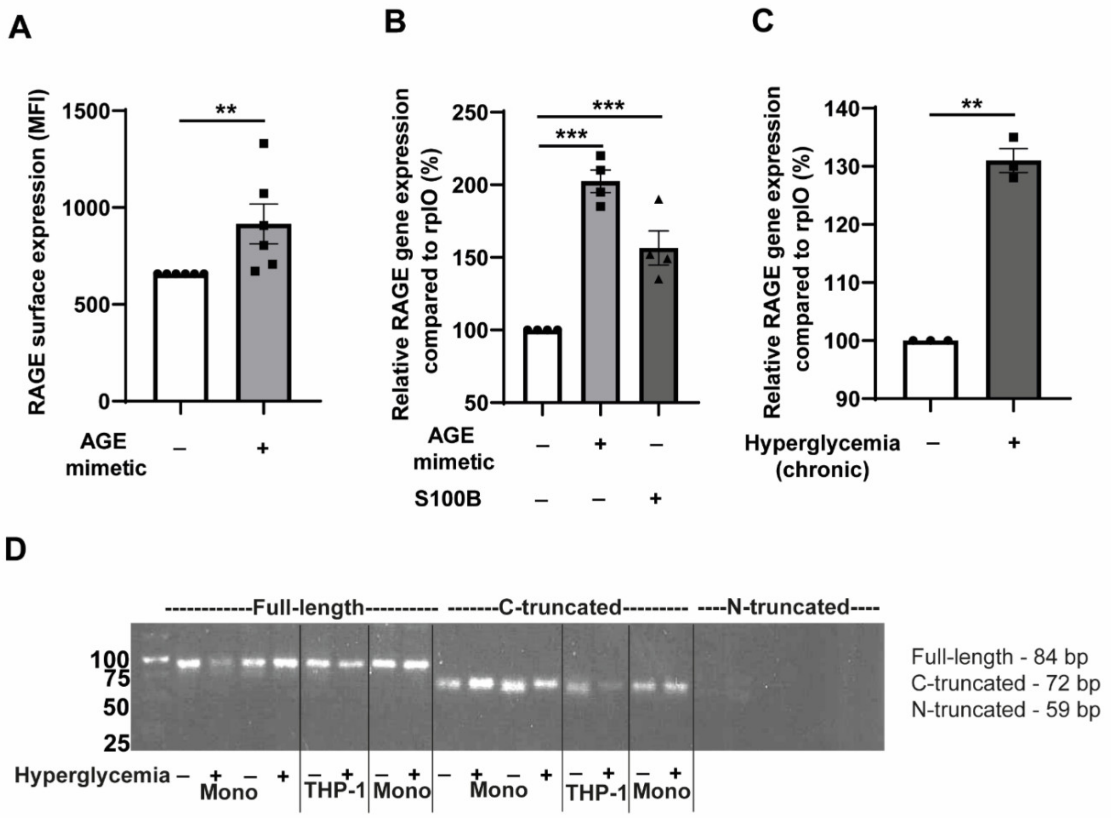

SUPPLEMENTARY FIGURE 5

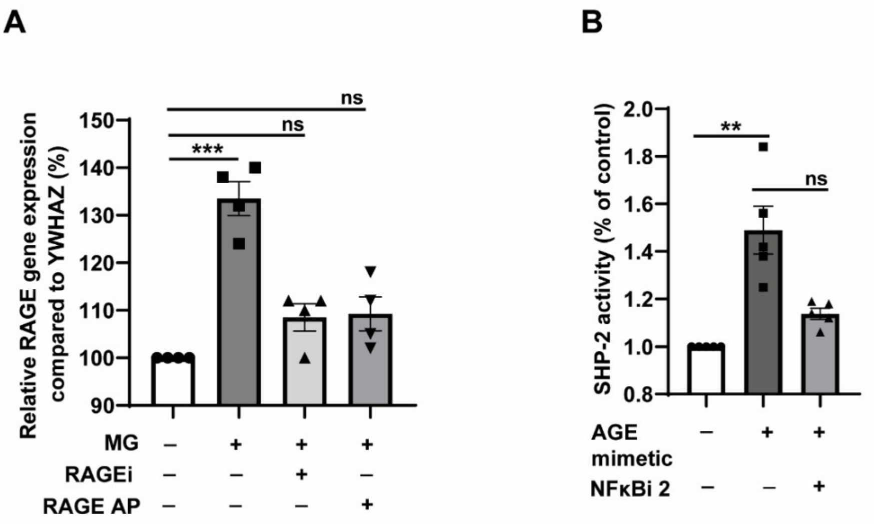

SUPPLEMENTARY FIGURE 6

Supplementary Figure Legends:

Supplementary Figure 1. SHP-2 controls the random migration of monocytes

(a) Relative expression of SHP-2 mRNA compared to HPRT. $Ly6C^+$ monocytes isolated from the bone marrow of T2DM, db/db mice (n=3) and non-T2DM wildtype mice (n=3) were used for the analysis. **(b)** SHP-2 phosphatase activity assay. Monocytes were treated with 300 µM MG for 24 hours Thereafter, the cells were lysed in anoxic conditions, and the phosphatase activity was measured in the SHP-2 immunoprecipitates using the Tyrosine Phosphatase Assay System (Promega). n=5. **(c)** Representative pictures of migrated monocytes with regard to the quantified data in Figure 1E indicating induction of random migration by Methylglyoxal. **(d)** Representative pictures of migrated monocytes with regard to data in Figure 1F revealing rescue of pro-migratory phenotype by the allosteric SHP-2 inhibitor SHP099. **(e)** Monocytes were treated with 300 µM MG for 24 hours, and the chemokinesis induction was analysed using Boyden chamber assays. Circles (untreated control) and squares (incubation with MG) indicate the migrated monocytes of each experiment in percentage relative to the mean of unstimulated control. n=4 **(f)** SHP-2 inhibitor-2, NSC 878777, with a distinct mode of action recapitulated the anti-migratory phenotype of MG-treated monocytes. Squares (incubation with MG) and triangles (incubation with MG and 400 nM NSC 878777) indicate migrated monocytes of each experiment in percentage in relation to the mean of unstimulated control. n=4 *p < 0,05, **p < 0.01, ***p < 0.001.

Supplementary Figure 2. Diabetic milieu-induced SHP-2 expression and THP-1 monocytic cell activation are reversible

(a) Experimental setup **(b)** Exposure of THP-1 monocytic cells with or without diabetic milieu (300 µM MG + 25 µg/ml AGE mimetic for 24 hrs. The cells were analysed for their ability to random migrate using transwell assays. n = 3. **(c)** Representative images of the randomly migrated THP-1 cells **(d)** Representative blots showing SHP-2 expression upregulation and **(e)** corresponding quantification. Expression of total-SHP-2 in relation to Vinculin **(f))** Exposure of THP-1 monocytic cells with or without diabetic milieu (300 µM MG + 25 µg/ml AGE mimetic for 24 hrs. After that the cells were rested for 6 days in a normal medium without MG or AGE mimetic. The cells were then analysed for their ability to random migrate using transwell assays. n = 3. **(g)** Representative images of the randomly migrated THP-1 cells **(h)** Representative blots showing SHP-2 expression upregulation and **(i)** corresponding quantification. Expression of total-SHP-2 in relation to Vinculin n = 3. *p < 0,05, **p < 0.01, ***p < 0.001.

Supplementary Figure 3. Dual inhibition of NFκB attenuates MG-, AGE-mimetic- and TNFα-induced SHP-2 expression

(a) Representative blots and **(b)** corresponding quantification. Expression of SHP-2 in relation to Vinculin. Exposure of Monocytes without (black bar) or with (grey bars) 300 µM MG for 24 hrs. Quantification of monocytes additionally treated with NFkBi 1 and NFkBi 2 is shown as the striped bar (1 µM Activation Inhibitor I, Calbiochem) and NFκBi 2 (5 µM NFκB Activation Inhibitor II, JSH23) or DMSO. n= 4. **(c)** Representative blots and **(d)** corresponding quantification. Expression of SHP-2 in relation to Vinculin. Exposure of Monocytes without (black bar) or with (grey bars) 200 µg/ml AGE-mimetic for 24 hrs. Quantification of monocytes additionally treated with NFkBi 1 and NFkBi 2 shown as the striped bar (1 µM Activation Inhibitor I, Calbiochem) and NFκBi 2 (5 µM NFκB Activation Inhibitor II, JSH23) or DMSO. n= 4. **(e)** Representative blots and **(f)** corresponding quantification. Expression of SHP-2 in relation to Vinculin. Exposure of Monocytes without (black bar) or with (grey bars) 10 µg/ml TNFα for 24 hrs. Quantification of monocytes additionally treated with NFkBi 1 and NFkBi 2 shown as the striped bar (1 µM Activation Inhibitor I, Calbiochem) and NFκBi 2 (5 µM NFκB Activation Inhibitor II, JSH23) or DMSO. n= 4. *p < 0,05, **p < 0.01, ***p < 0.001.

Supplementary Figure 4. MG-induced SHP-2 expression is driven by NFκB but it does not contribute to MG-induced inflammation induction

(a) Representative blots and **(b)** corresponding quantification. Expression of SHP-2 in relation to ß-actin. Exposure of Monocytes without (black bar) or with (grey bars) 300 µM MG for 24 hrs. Quantification of monocytes additionally treated with NFkB-inhibitor-1 is shown as a striped bar (1 µM Activation Inhibitor I, Calbiochem). n= 4. **(c)** Representative blots and **(d)** corresponding quantification. Expression of total-p65 in relation to Vinculin. Exposure of Monocytes without (black bar) or with (grey bars) 300 µM MG for 24 hrs. Striped and dotted bars indicate knockdown efficiency after transfection with siRNA against NFkB-p65. n = 5. **(e, f, g)** Monocytes were treated with 300 µM MG for 24 hours in the presence or absence of 700 nM SHP-2i (SHP099). After that, the supernatants were collected and the levels of TNFa, IL-6 and IL-1β were detected using sandwich ELISA. n=6. *p < 0,05, **p < 0.01, ***p < 0.001.

Supplementary Figure 5. Expression of RAGE in monocytes and its splice variants

(a) FACS analysis of the surface expression of RAGE. Exposure of monocytes without (white bar) or with (grey bar) 150 µg/ml AGE-BSA (AGE-mimetic) for 24 hrs. Cells were then analysed for the surface expression of RAGE. n=6. **(b)** Relative expression of RAGE mRNA compared to rplO. Primary monocytes were exposed to 150 µg/ml AGE-BSA (AGE-mimetic) or 50 µg/ml S100B. After that the cells were lysed, and the expression of RAGE mRNA was detected using RT-qPCR. n=4. **(c)** THP-1 monocytic cell line was exposed to hyperglycemic

conditions continuously for 5 days. On the 6th day, the cells were lysed, and the expression of RAGE mRNA was detected using RT-qPCR. n=3. **(d)** Splice variant detection of RAGE in THP-1 cells and primary monocytes exposed to hyperglycemic conditions using RT-PCR. **p < 0.01, ***p < 0.001.

Supplementary Figure 6. The RAGE-NFκB axis controls SHP-2 expression activity

(a) Relative expression of SHP-2 mRNA compared to YWHAZ. Monocytes were treated with 300 μM MG for 24 hours. In addition, the MG-treated cells were additionally treated with RAGE-inhibitor (RAGEi, 200 nM FPS-ZM1, Calbiochem) or RAGE-antagonist peptide (RAGE AP, 2 μM, Calbiochem) or DMSO. Thereafter the cells were lysed, and the expression of RAGE mRNA was detected using RT-qPCR. n=4. (**b**) SHP-2 phosphatase activity assay. Monocytes were treated with 150 μg/ml AGE-BSA (AGE-mimetic) for 24 hours. In addition, the AGE-BSA treated cells were exposed to 5 μM NFκB Activation Inhibitor II, NFκBi 2, JSH23 or DMSO. After that, the cells were lysed in anoxic conditions and the phosphatase activity was measured in the SHP-2 immunoprecipitates using the Tyrosine Phosphatase Assay System (Promega). n=5.*p < 0,05, **p < 0.01, ***p < 0.001.

Table S1. Clinical characteristics of the non-T2DM and T2DM individuals used in the study.

	Non-DM	T2 DM	significance
N	12	12	
Age (years)	56±17.8	63±7.2	n.s.
Sex (male/female)	9/3	8/4	n.s.
BMI (kg/m^2)	26.5±3.2	36.9±4.9	<0.001
HbA1c (%)	5.33±0.25	8.64±1.02	<0.001
Glucose (mmol/L)	5.06±0.44	12.7±4.75	<0.001
Smoking (yes/no)	1/11	1/11	n.s.
Hypercholesterolaemia (yes/no)	6/6	0/12	0.006

Table 2 RT-qPCR primer sequences used in the study.

**Gene**	**Forward primer sequence (5' -3')**	**Reverse primer sequence (5' -3')**
**hSHP-2**	CCCACAATCAAGATTCAGAACA CT	GCCCGTGATGTTCCATGTAA
**hRAGE**	AGGAGGAAGAGGAGGAGCGT	TGGCAAGGTGGGGTTATACAG

**hTNFa**	CAGAGGGCCTGTACCTCATC	GGAAGACCCCTCCCAGATAG
**hrplO**	AATCTCCAGGGGCACCATT	CGCTGGCTCCCACTTTGT
**hYWHAZ**	ACTTTTGGTACATTGTGGCTTCAA	CCGCCAGGACAAACCAGTAT
**mSHP-2**	ACCCTGTGCAGAAATGAGGG	CCGTGGGCTCATCTGAAACT
**mRAGE**	GGACCCTTAGCTGGCACTTAGA	GAGTCCCGTCTCA-GGGTGTCT
**mHprt**	GCAGTACAGCCCCAAAATGG	AACAAAGTCTGGCCTGTATCCAA

Table S3. Splice variant detection of RAGE: RT-PCR primers used.

Gene	Forward primer sequence (5' -3')	Reverse primer sequence (5' -3')
**Full RAGE**	GCTGTCAGCATCAGCATCAT	AGGGCCAGGGCTAGAGTTC
**C-trunc. RAGE**	GCTGTCAGCATCAGCATCAT	CCCTGACTTTATCAAACCCC
**N- trunc. RAGE**	AGTGCCTTTCAAGGTCCCTC	GGAAAGGGAATGAGGGCTAAC
